# Barrier‐Assisted Plasma Doping for Spatially Selective Resistance Engineering in MoS_2_ Transistors

**DOI:** 10.1002/adma.74389

**Published:** 2026-08-01

**Authors:** Inseong Lee, Joonho Park, Seungsun Yoo, Seunghyun Yu, Mingu Kang, Seohak Park, Min Kyu Lee, Hyeongjin Lim, Dongyoung Kim, Kunwoo Roh, Sejin Kim, Kihyuk Kim, Wonbae Ahn, Kibum Kang, Yong‐Hoon Kim, Sung‐Yool Choi

**Affiliations:** ^1^ School of Electrical Engineering Korea Advanced Institute of Science and Technology (KAIST) Daejeon Republic of Korea; ^2^ Graduate School of Semiconductor Technology Korea Advanced Institute of Science and Technology (KAIST) Daejeon Republic of Korea; ^3^ Department of Materials Science and Engineering Korea Advanced Institute of Science and Technology (KAIST) Daejeon Republic of Korea

**Keywords:** 2D transistor, degenerate doping, enhancement‐mode, molybdenum disulfide, NH_3_ plasma, protective dielectric stack

## Abstract

Atomically thin transition‐metal dichalcogenides (TMDs) are promising channel materials for low‐power logic. However, the absence of scalable and region‐selective doping techniques leads to excessive local resistances that hinder the technological readiness of 2D transistors. Here, a barrier‐assisted NH_3_ plasma process is demonstrated that enables degenerate n‐type doping of monolayer MoS_2_ while preserving its crystallinity. The ultrathin pV3D3/Al_2_O_3_ dielectric stack not only blocks plasma‐induced damage but also functions as a chemical filter that permits NH_x_ radicals to diffuse through. Through this doping process, an electron density of 4.3 × 10^13^ cm^−2^ is achieved, yielding a contact resistance of 1.45 kΩ·µm. Density functional calculations show that NH_2_ radicals adsorbed on the pristine MoS_2_ surface are the main source of n‐type doping while NH radicals can heal S‐vacancy defects. Leveraging the spatial selectivity of this approach, mobility and on‐current are enhanced by 5.8‐fold with negligible threshold‐voltage shift. Extension‐region activation further suppresses series resistance, increasing the on‐current by 260‐fold (*V*_DS_ = 0.05 V) while maintaining enhancement‐mode operation. These findings establish barrier‐assisted NH_3_ plasma doping as a promising approach for enabling high‐performance n‐type 2D transistors and advancing future energy‐efficient 2D CMOS technology.

## Graphical abstract

Barrier‐assisted NH_3_ plasma doping enables damage‐free, spatially selective carrier modulation in monolayer MoS_2_ transistors. An ultrathin pV3D3/Al_2_O_3_ protective‐dielectric stack acts as a chemical filter that allows NH_x_ radicals to reach the MoS_2_ surface while blocking plasma damage. This process achieves a high electron concentration of 4.3 × 10^13^ cm^−2^, enabling effective resistance engineering in 2D transistors.

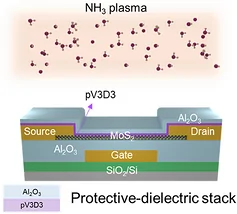

## 1 Introduction

Two‐dimensional (2D) transition metal dichalcogenides (TMDs), such as MoS_2_ have attracted considerable interest as channel materials for next‐generation electronics due to their atomic‐scale thickness, excellent electrostatic gate control, and resistance to short‐channel effects [1, 2, 3]. These attributes make 2D FETs attractive for both device downscaling and advanced integration schemes, including monolithic three‐dimensional (3D) architectures [4]. Despite these advantages, the practical performance of 2D transistors remains limited by large contact and series resistances in the extension regions, which critically degrade current drivability and switching speed. A primary factor underlying high contact resistance is strong Fermi level pinning at the metal‐semiconductor interface, resulting in substantial Schottky barrier heights that hinder efficient carrier injection [5, 6, 7]. In underlapped top‐gate devices, the intrinsically low carrier density in the extension regions becomes a dominant source of series resistance, since these areas are not electrostatically modulated by the gate [8, 9]. Therefore, an additional doping process is indispensable for enabling low resistance, high‐performance switching in 2D transistors. However, unlike silicon, 2D materials cannot rely on conventional ion implantation or diffusion, making it difficult to achieve scalable and selective doping for complementary metal‐oxide‐semiconductor (CMOS) integration [10, 11, 12]. As a result, alternative approaches molecular, dielectric‐assisted, and plasma‐based doping strategies have been pursued, yet each suffers from key limitations in selectivity and scalability [13, 14, 15, 16]. Surface molecular doping can achieve high carrier densities without structural degradation; however, it generally lacks spatial selectivity and uniformity over large areas, limiting its practical applicability [17, 18]. Dielectric‐assisted doping using defective oxides or nitrides offers improved stability, but the additional dielectric layers undesirably increase overall device thickness and introduce parasitic capacitance, potentially degrading switching performance and hindering further scaling [19, 20]. Plasma‐based doping, which in principle enables scalable and spatially selective carrier modulation, has been widely investigated as an effective approach for tuning the carrier densities in 2D semiconductors [21]. Among various plasma‐based methods, NH_3_ plasma can serve as an effective n‐doping approach by supplying –NH_x_ radicals that can chemically bind to the MoS_2_ surface and act as electron donors [22]. However, direct exposure of MoS_2_ to plasma often induces permanent structural damage, leading to severely degraded device performance [23, 24, 25, 26]. To address these limitations, we propose a barrier‐assisted NH_3_ plasma doping process that enables high‐density, spatially confined n‐type doping of MoS_2_ without compromising crystallinity. The critical challenge is to introduce desired dopant species, such as –NH_2_, onto the MoS_2_ surface without causing lattice damage. If the barrier is too thick, it blocks dopant species from reaching the underlying MoS_2_ and suppresses the intended doping, whereas if it is too thin, uniform barrier deposition cannot be achieved, leaving pinholes and incomplete protection.

Our approach employs a bilayer protective‐dielectric stack consisting of polymeric poly(1,3,5‐trimethyl‐1,3,5‐trivinyl cyclotrisiloxane) (pV3D3) grown by initiated chemical vapor deposition (iCVD) and of ALD‐grown Al_2_O_3_. The pV3D3 layer ensures uniform dielectric coverage and suppresses interfacial traps, whereas the ultrathin Al_2_O_3_ overlayer protects the underlying lattice from plasma‐induced damage. This unique bilayer design permits chemical doping through the barrier while preventing plasma‐induced etching, effectively acting as a precise chemical filter that both preserves the MoS_2_ interface quality and enables controlled, damage‐free permeation of NH_x_ species essential for doping. Using this method, we demonstrate degenerately n‐doped MoS_2_ with carrier densities approaching 4.3 × 10^13^ cm^−2^. First‐principles calculations reveal that, while NH_2_ radicals adsorbed on the MoS_2_ surface act as the primary source of n‐type doping, NH radicals concurrently passivate S vacancy defects. This dual‐functional mechanism provides a theoretical basis for the experimentally observed transition from defect‐dominated hopping transport in untreated MoS_2_ to high‐quality band‐like transport in the Al_2_O_3_‐protected pV3D3‐MoS_2_. We further exploit the spatial selectivity of the process to target doping at the channel, contact, and extension regions in back‐gated, overlapped top‐gated, and underlapped top‐gated architectures. Selective contact doping substantially lowers the effective Schottky barrier height (from 226.7 to 73.7 meV), enabling improved carrier injection and a 5.8‐fold mobility enhancement without threshold‐voltage shift. Extension‐region doping suppresses parasitic series resistance in underlapped devices, yielding a 260‐fold on‐current increase at low drain bias (0.05 V) while preserving enhancement‐mode operation. These results demonstrate that barrier‐assisted plasma doping enables spatially selective resistance engineering in 2D transistors, providing a scalable pathway toward high‐performance and energy‐efficient CMOS integration.

## 2 Results

### 2.1 Mechanism of Barrier‐Assisted NH_3_ Plasma Doping in MoS_2_

To achieve a uniform dielectric stack on MoS_2_, we first employed the iCVD process to deposit an ultrathin pV3D3 layer. The pV3D3 layer promotes uniform, pinhole‐free coverage on the chemically inert MoS_2_ surface and reduces interfacial trap states, thereby improving both dielectric nucleation and interface quality [27]. However, a single pV3D3 layer does not provide sufficient protection against plasma‐induced damage to MoS_2_ (Figure S1); therefore, an additional ultrathin Al_2_O_3_ overlayer is incorporated to reinforce the barrier functionality. This Al_2_O_3_ layer suppresses plasma‐induced etching of the pV3D3 while simultaneously allowing controlled diffusion of reactive radicals through the barrier [28]. In addition, this bilayer scheme yields superior surface roughness and film uniformity compared to a single Al_2_O_3_ layer (Figure S2). Consequently, the bilayer protective‐dielectric (PD) stack was formed by combining 2 nm of iCVD‐grown pV3D3 with 2 nm of ALD‐grown Al_2_O_3_. This stack serves a dual role: physical protection from plasma‐induced damage while enabling chemical access of reactive NH_3_‐derived radicals to the underlying MoS_2_. (Figure 1a)

**FIGURE 1:**
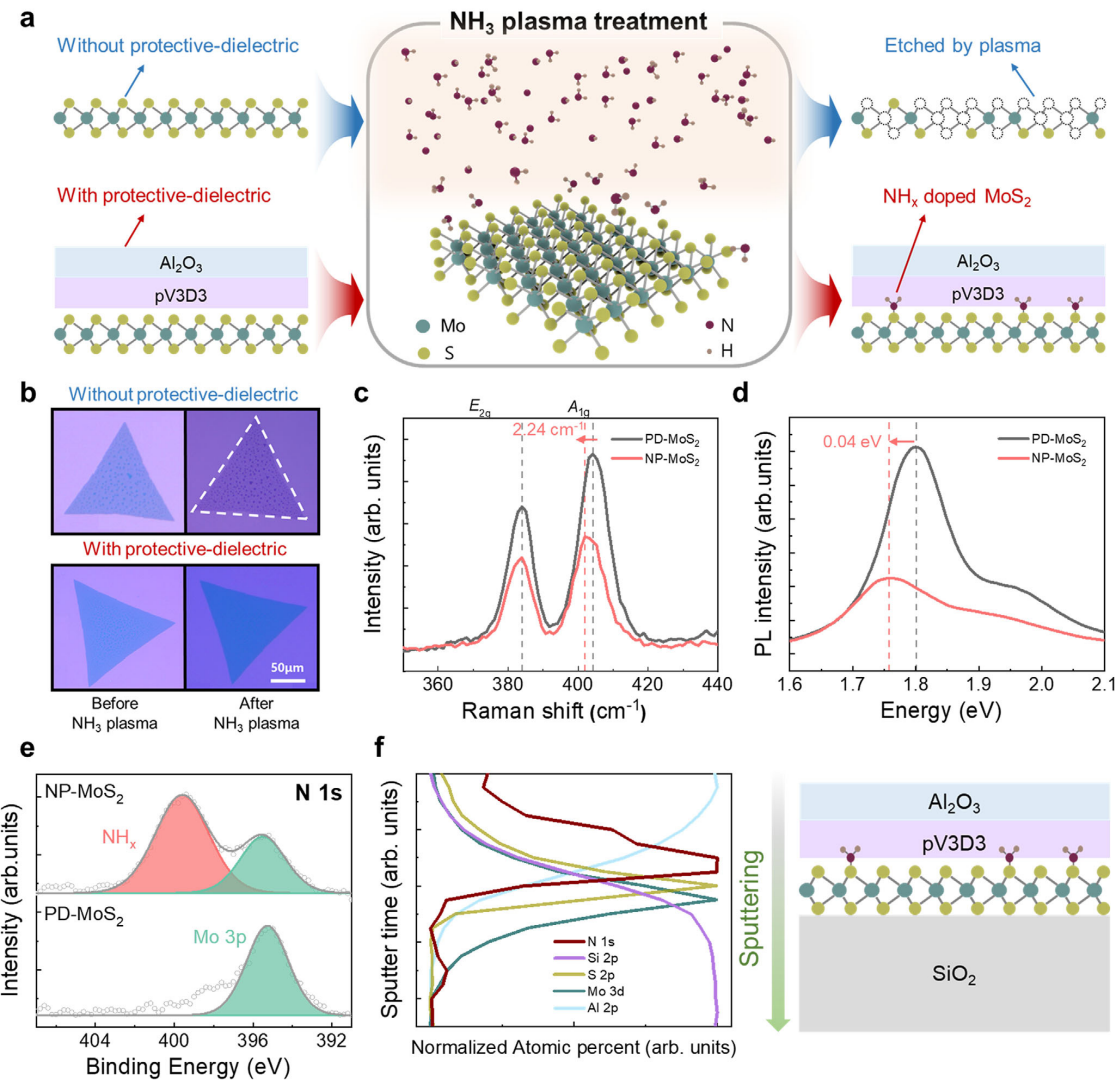
Mechanism of protective‐dielectric‐assisted NH_3_ plasma doping in monolayer MoS_2_. (a) Schematic illustration of NH_3_ plasma exposure on monolayer MoS_2_ without (top) and with (bottom) an ultrathin 2 nm Al_2_O_3_/2 nm pV3D3 protective‐dielectric (PD). (b) Optical microscopy (OM) images of MoS_2_ and PD‐MoS_2_ after NH_3_ plasma. (c–e) Raman, photoluminescence (PL), and N 1s X‐ray photoelectron spectroscopy (XPS) characterizations of MoS_2_ before (black) and after NH_3_ plasma treatment through the PD (red). (f) Normalized XPS depth profile showing N 1s localized at the MoS_2_ surface above S 2p/Mo 3d signals.

To validate the efficacy of this barrier design, we compared pristine MoS_2_ and PD stack‐deposited MoS_2_ (PD‐MoS_2_) subjected to identical NH_3_ plasma treatments. Figure 1b presents optical microscopy images of MoS_2_ after plasma treatment. Even with mild plasma exposure, pristine MoS_2_ suffers from pronounced etching and structural degradation. The resulting loss of crystallinity is unambiguously confirmed by Raman spectroscopy (Figure S3). In contrast, PD‐MoS_2_ retains a well‐defined morphology even after 240 min of plasma treatment, and the presence of sharp Raman peaks further confirms that its crystallinity is well‐preserved, indicating that our dielectric stack effectively suppresses plasma‐induced damage.

To confirm that doping proceeds despite the physical barrier, we performed Raman spectroscopy as shown in Figure 1c. The E_2g_ mode, corresponding to the in‐plane vibrational mode, is primarily sensitive to strain, whereas the A_1g_ mode, associated with the out‐of‐plane vibrational mode, is predominantly affected by doping [29, 30]. Raman spectra of NH_3_ plasma‐treated MoS_2_ (NP‐MoS_2_) exhibit a distinct 2.24 cm^−1^ red shift of the A_1g_ peak, with negligible shift in the E_2g_ peak. These changes indicate enhanced electron density without inducing strain or major structural distortion, suggesting that doping occurs without introducing unnecessary structural perturbations [31, 32]. Consistent with these findings, the photoluminescence (PL) spectra in Figure 1d reveal that, after NH_3_ plasma treatment, the main emission peak (∼1.8 eV) undergoes a clear red shift accompanied by a pronounced reduction in intensity, providing strong evidence of substantial n‐type doping [33, 34, 35]. These optical characterizations collectively demonstrate that electron density in MoS_2_ can be effectively increased while preserving crystallinity, thereby validating the effectiveness of the PD stack.

To investigate the origin of the observed n‐type doping, we employed X‐ray photoelectron spectroscopy (XPS) to analyze the surface states of MoS_2_ before and after NH_3_ plasma treatment, as shown in Figure 1e. The most prominent difference is the emergence of a new peak in the N 1s region. PD‐MoS_2_ shows only the Mo 3p signal in the N 1s range (∼395.5 eV), whereas NP‐MoS_2_ exhibits a prominent new peak near 399.5 eV, characteristic of –NH_x_ species such as –NH and –NH_2_ [36, 37]. Concurrently, the Mo 3d spectrum of NP‐MoS_2_ exhibits a clear blue shift, indicative of increased electron density and the onset of n‐type doping (Figure S4). This observation suggests that the newly detected –NH_x_ peak in the N 1s spectrum is likely the primary origin of this n‐doping behavior. To pinpoint the location of the newly formed –NH_x_ bonds and gain insight into the doping mechanism, we performed XPS depth profiling. The analysis revealed that the N 1s peak is predominantly localized on the MoS_2_ surface, followed sequentially by the S 2p and Mo 3d peaks in the underlying layers (Figure 1f). This indicates that –NH_x_ species selectively bind to the outermost atomic layer of MoS_2_ while preserving the underlying lattice in an undamaged state. EDS mapping further verifies that, despite the presence of the PD stack, nitrogen atoms are successfully localized on the MoS_2_ surface (Figure S5). Collectively, these results demonstrate that NH_3_ plasma treatment with a PD stack enables NH_x_ radicals to penetrate the dielectric layer and bind to the MoS_2_ surface while preserving the integrity of the underlying lattice, thereby enabling controlled electron doping without structural degradation. Furthermore, the clear polarity conversion from p‐type to n‐type transport observed in a monolayer WSe_2_ FET after NH_3_ plasma treatment suggests that this barrier‐assisted doping strategy may be extendable beyond MoS_2_ to other TMD semiconductors (Figure S6).

### 2.2 Dual Role of NH_x_ Radicals in Doping and Defect Passivation of MoS_2_

To elucidate the microscopic origin of the n‐type doping under NH_3_ plasma treatment of PD‐MoS_2_, we performed density functional theory (DFT) calculations to analyze the energetics and electronic structure of NH_x_‐adsorbed MoS_2_ (NH_x_/MoS_2_). A 5 × 5 unit cell model of MoS_2_ was employed, in which adsorption of a single NH_2_ radical (see below) corresponds to a doping concentration of 4.7 × 10^13^ cm^−2^, comparable to the experimentally observed doping level (Figure S7) [38, 39, 40]. We first examined the formation energies of ${\mathrm{NH}}_{x}^{*}$ (*x*  = 0, 1, 2) species on a pristine MoS_2_ surface generated from N and H radicals that permeate through the PD stack. The formation energy was defined as [38–40]

(1) $$E_{\mathrm{form}}=E_{NH_{x}/\mathrm{Mo}S_{2}}\;-\left(E_{\mathrm{Mo}S_{2}}+E_{N}+xE_{H}\;\right)$$

where $E_{NH_{x}/\mathrm{Mo}S_{2}}$, $E_{\mathrm{Mo}S_{2}}$, *E*_N_, and *E*_H_ are the total energies of the NH_*x*_‐adsorbed MoS_2_, pristine MoS_2_, isolated N radical, and isolated H radical, respectively. The calculated reaction pathways presented in Figure 2a (see also Table S1) include: (i) stepwise adsorption of N and H atoms on a surface S site (black dashed lines) (ii) gas‐phase formation of NH_2_ followed by adsorption (blue dashed lines), and (iii) the resulting adsorption energies of NH_x_ species (red dashed lines; see also Table S2). In all scenarios, ${\mathrm{NH}}_{2}^{*}$ is the most energetically favorable configuration. Transient N*and NH* species are exothermically hydrogenated to ${\mathrm{NH}}_{2}^{*}$, whereas further hydrogenation of ${\mathrm{NH}}_{2}^{*}$ leads to NH_3_ formation and desorption, indicating that ${\mathrm{NH}}_{2}^{*}$ is the stable surface dopant species responsible for charge transfer.

**FIGURE 2:**
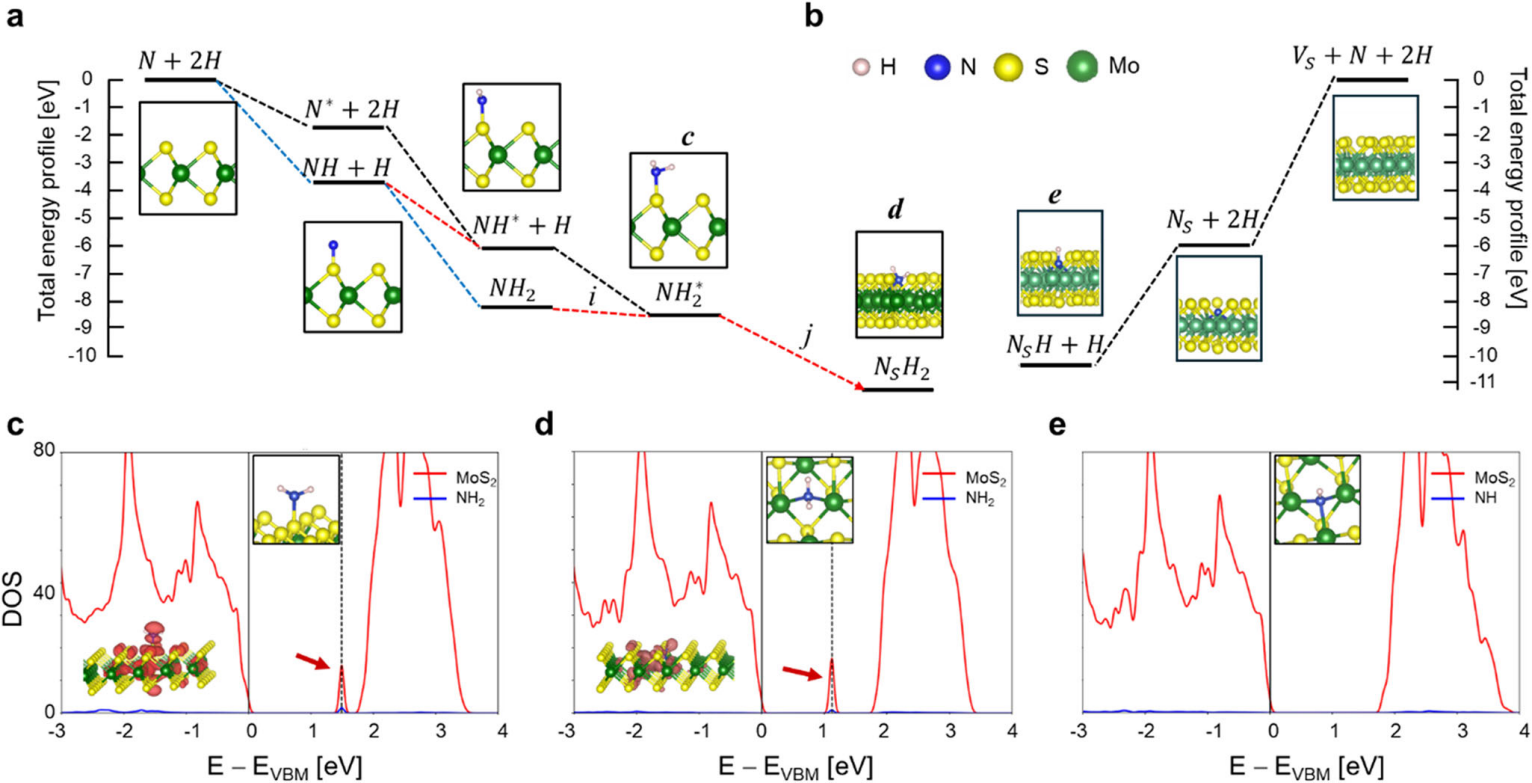
DFT investigations of NH_x_ adsorption on pristine and V_S_‐containing MoS_2_. (a,b) Calculated reaction‐energy profiles for the NH_x_ radical formation and adsorption (a) on the pristine MoS_2_ surface and (b) on the MoS_2_ containing a sulfur vacancy (V_S_). (c–e) PDOS of (c) NH_2_‐adsorbed MoS_2_ (${\mathrm{NH}}_{2}^{*}$/MoS_2_), (d) NH_2_‐passivated V_S_(N_S_H_2_/MoS_2_), and (e) NH‐passivated V_S_ (N_S_H/MoS_2_), respectively. The insets show the partial charge densities of the in‐gap states marked by red arrows. For NH_2_‐adsorbed MoS_2_ and N_S_H_2_/MoS_2_ produce shallow donor states and deep trap states, respectively, whereas N_S_H/MoS_2_ exhibits the recovery of pristine MoS_2_bandgap.

The electronic structure of ${\mathrm{NH}}_{2}^{*}$/MoS_2_ is shown in Figure 2c. The projected density of states (PDOS) [39, 40] reveals donor states introduced below the conduction band minimum (CBM), accompanied by the Fermi‐level shift toward CBM, consistent with n‐type doping. The inset showing the corresponding partial charge density indicates that the donor states are primarily delocalized over the MoS_2_ lattice rather than localized on the NH_2_ moiety, confirming substantial electron transfer from NH_2_ to the MoS_2_ conduction band. Spin‐polarized calculations (Figure S8) show a minor exchange splitting (∼0.03 eV), originating from the odd‐electron NH_2_ radical, while the MoS_2_ substrate remains essentially non‐magnetic.

Given that fabrication‐induced defects are unavoidable in CVD‐grown MoS_2_, we further investigated dopant‐defect interactions by considering a V_S_. The formation energies similarly calculated for this case are summarized in Figure 2b (see also Figure S9) [41, 42]. When NH_2_ is captured at the V_S_ site, the optimized NH_2_/MoS_2_‐V_S_ (N_S_H_2_/MoS_2_) structure relaxed into a double‐bridge configuration in which the N atom is bonded to two H atoms and two neighboring Mo atoms, reflecting the sp^3^‐like bonding nature of N. This configuration is lower in energy than the NH_2_ adsorption on an MoS_2_ on‐top S site (${\mathrm{NH}}_{2}^{*}$/MoS_2_) by 2.78 eV, indicating that V_S_ sites will act as strong trapping centers for mobile ${\mathrm{NH}}_{2}^{*}$ species and thereby suppress their long‐range interfacial migration. The corresponding PDOS of N_S_H_2_/MoS_2_ (Figure 2d) show deep donor states, indicationg that NH_2_ trapped at the V_S_ site will act as scattering centers.

We then discuss the NH‐passivated V_S_ configuration (N_S_H/MoS_2_). As shown in Figure 2b, N occupies the V_S_ site with a significantly larger binding energy (‐6.19 eV) compared to its adsorption on an MoS_2_ surface S site (−1.70 eV). We found that subsequent hydrogenation further stabilizes the S vacancy‐passivating NH configuration (N_S_H/MoS_2_, −4.07 eV) but in this scenario the second hydrogenation to N_S_H_2_/MoS_2_ is prohibited due to the hydrogen evolution mechanism to H_2_ + N_S_/MoS_2_. Then, the N_S_H/MoS_2_ PDOS demonstrates restoration of the pristine MoS_2_ bandgap or the elimination of vacancy‐induced mid‐gap states (Figure 2e; Figure S9). Temperature‐dependent PL measurements further support this defect‐passivation mechanism (Figure S10). After NH_3_ plasma treatment, the defect‐related localized emission peak, commonly referred to as the L peak, was strongly suppressed, as evidenced by the reduced *I*_L_/*I*_A_ ratio over the measured temperature range. In addition, the A peak exhibited a red shift after NH_3_ plasma treatment, consistent with increased electron density and n‐type doping. These temperature‐dependent PL results provide additional optical evidence that NH_3_ plasma treatment suppresses V_S_‐related localized defect emission while inducing electron doping in MoS_2_. Collectively, these results indicate that NH_3_ plasma treatment of PD‐MoS_2_ induces robust n‐type doping primarily through NH_2_ adsorption on a surface S site, while simultaneously healing sulfur vacancies via NH passivation, thereby enhancing carrier density and allowing band‐like transport without interruption by deep defect states.

### 2.3 Channel and Contact Resistance Engineering in NH_3_ Plasma‐doped MoS_2_ FETs

We next investigated how such chemically induced carrier accumulation manifests in transistor‐level device operation. We first fabricated back‐gated (BG) devices employing a 15 nm Al_2_O_3_ layer as the gate dielectric. Subsequently, our PD stack was deposited on top of the MoS_2_ channel, as illustrated in Figure 3a. NH_3_ plasma was then selectively applied to the channel region under mild plasma conditions (5 W), and the transfer characteristics were measured after cumulative exposure times ranging from 20 to 240 min to examine time‐dependent doping effects. (Figure 3b) The gray curve in Figure 3b corresponds to the transfer characteristics prior to plasma treatment, showing that the devices retain a high on/off current ratio even after deposition of the PD stack. This confirms that the PD stack deposition alone does not induce significant doping, and that the observed n‐doping effect originates solely from the subsequent plasma process. Upon increasing the plasma exposure time, a progressive negative shift in the threshold‐voltage (V_TH_) and a noticeable increase in the on‐current were observed. Notably, after more than 180 min of NH_3_ plasma exposure, the on/off ratio collapsed to below 2 while the on‐current increased by more than seventeen‐fold, confirming the emergence of strongly degenerate n‐type behavior. To quantify the induced carrier concentration, the two‐dimensional carrier concentration (n_2D_) was extracted from the V_TH_ shift using the relation *n*_2D_ = *C*_ox_Δ*V*_TH_/q (Figure 3c). Even after only 20 min of plasma exposure, the electron concentration exceeded 1 × 10^13^ cm^−2^, and it eventually saturated at 4.3 × 10^13^ cm^−2^ after 180 min of treatment. These results demonstrate that the carrier density can be deliberately tuned simply by adjusting the plasma exposure time, indicating that the doping level is externally controllable. Based on these observations, a plasma treatment duration of 180 min was adopted as the standard condition for all subsequent experiments. This controllable doping behavior was further supported by statistical analysis of 20 BG devices, in which the distributions of V_TH_ and field‐effect mobility consistently confirmed the NH_3_ plasma induced n‐type doping effect through the PD stack (Figure S11 and Table S3).

**FIGURE 3:**
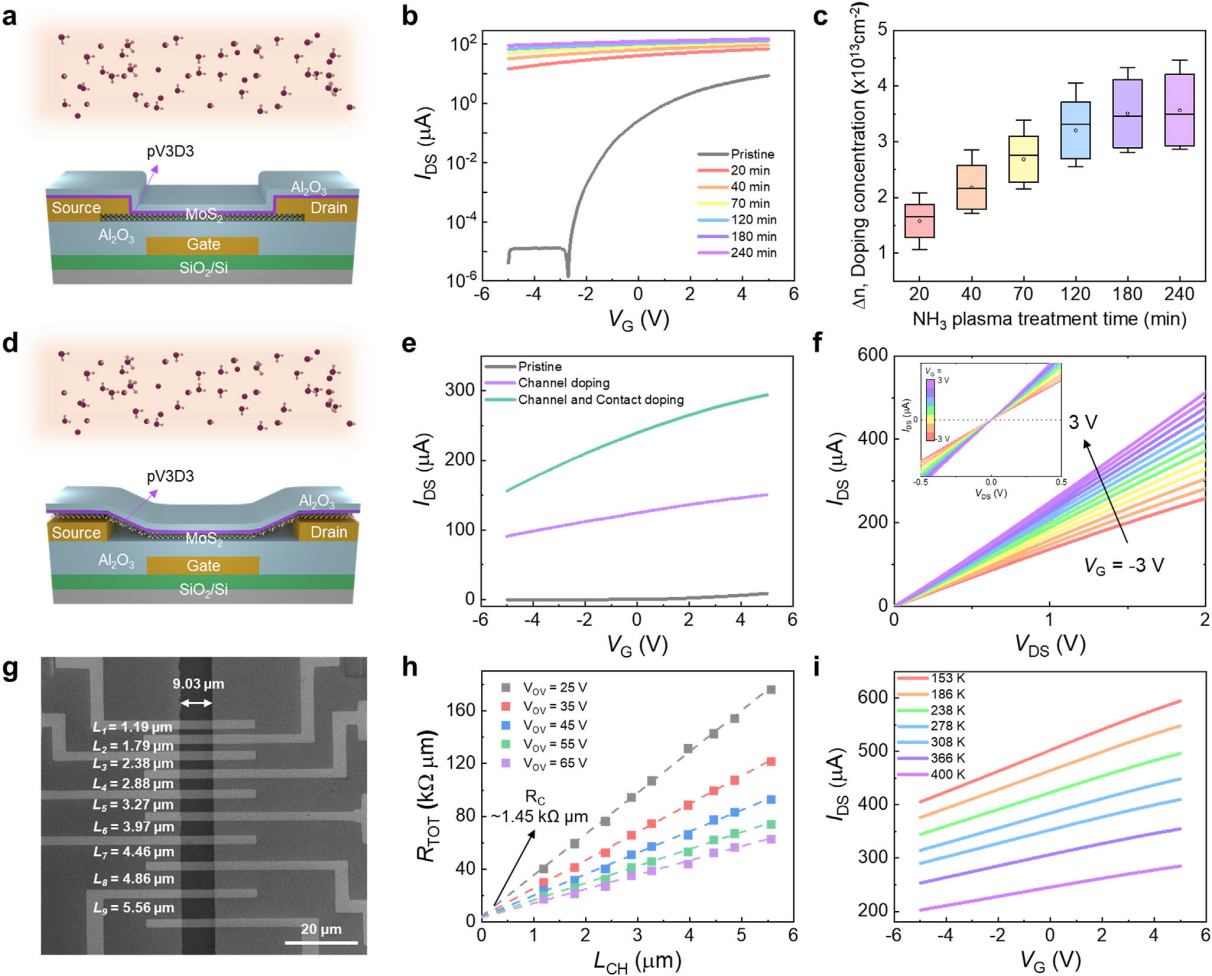
Localized NH_3_ plasma doping of MoS_2_ FETs for channel and contact resistance engineering. (a) Schematic of back‐gated MoS_2_ FETs used to assess channel‐selective plasma doping under a PD. (b) Transfer curves of devices subjected to increasing plasma exposure durations. (c) Extracted 2D carrier concentration as a function of plasma exposure time. (d) Schematic of a modified device architecture in which both the channel and source/drain contact regions are exposed to plasma. (e) Transfer curves of undoped, channel‐only doped, and dual‐region doped devices. (f) Output characteristics of dual‐region‐doped devices; the inset highlights the linearity in the low‐bias regime. (g) SEM image of the TLM structure. h Total resistance as a function of channel length extracted from the TLM structure. (i) Temperature‐dependent transfer curves of doped devices.

To further enhance carrier injection and reduce the total device resistance beyond what is achievable with channel‐only doping, we expanded the doping region to encompass not only the gated channel but also the source/drain contact regions. This strategy was enabled by a slight architectural modification in which monolayer MoS_2_ was transferred onto pre‐patterned metal electrodes, thereby allowing the contact regions to be directly exposed to the NH_3_ plasma. The reconstructed device configuration is shown in Figure 3d, and the corresponding transfer curves before and after dual‐region (channel and contact) doping are presented in Figure 3e. In comparison with channel‐only doped devices (Figure 3a), dual‐region doped devices exhibited more than a two‐fold enhancement in both on‐current and transconductance, demonstrating that simultaneous doping of the channel and contacts more effectively reduces the overall device resistance. These results confirm that the proposed barrier‐assisted NH_3_ plasma process enables controlled electron doping in both regions, yielding a substantial improvement in carrier injection efficiency. Figure 3f shows that the dual‐region doped device exhibits highly linear output characteristics across the full bias range. This linear behavior is attributed to ohmic‐like carrier injection, which arises from substantial thinning of the Schottky barrier due to the increased electron concentration on the contact region. Separate stability measurements on NH_3_ plasma‐doped BG MoS_2_ FETs further confirmed the robustness of the doping effect under ambient exposure, 250°C annealing, and gate‐bias stress, showing negligible changes in the extracted doping level and on‐current (Figure S12).

In order to reliably quantify the reduced channel and contact resistance values after doping, transmission line method (TLM) structures with nine different channel lengths were fabricated. The SEM image of the fabricated TLM pattern is shown in Figure 3g. From the linear fit of total resistance versus channel length, we extracted a low contact resistance of 1.45 kΩ·µm (Figure 3h), which is substantially reduced from the undoped value of 47 kΩ·µm. These results demonstrate that our barrier‐assisted process achieves substantial carrier accumulation without structural degradation typically associated with direct plasma exposure.

The fundamental change in electronic quality after the NH_3_ plasma treatment is further elucidated by temperature‐dependent electrical measurements. Prior to doping, the devices exhibit a “hopping transport” mechanism as shown in Figure S13, where on‐current increases with temperature—a characteristic behavior of insulating states dominated by V_S_ and interfacial defect‐induced localized states [43]. In stark contrast, the doped device exhibits a reversal of this trend: as shown in Figure 3i, the on‐current decreases as temperature increases across the entire gate bias range.

This temperature‐inverted behavior is a hallmark of “band‐like transport” in highly degenerate semiconductors. Notably, our DFT investigations provided the microscopic explanation for this transition. While NH_2_ adsorption introduces the necessary donor states for n‐type doping, NH radicals concurrently passivate V_S_ by occupying vacant sites, thereby eliminating mid‐gap trap states and restoring the pristine bandgap. This dual‐functional mechanism simultaneous charge donation and defect healing fundamentally shifts the transport mechanism, enabling band‐like transport in MoS_2_ [42, 44]

### 2.4 Schottky Barrier Engineering via Contact‐Selective NH_3_ Plasma Doping

Building upon the previously demonstrated channel‐level doping results, we next extended our barrier‐assisted NH_3_ plasma doping strategy to top‐gated MoS_2_ FETs. The primary objective was to selectively enhance the contact regions while preserving the low‐power, enhancement‐mode operation of the device. Figure 4a schematically illustrates the device architecture and the corresponding doping procedure. Our devices adopt a source/drain‐first, top‐gate configuration, in which MoS_2_ is transferred onto pre‐patterned Au source and drain electrodes. This architecture inherently mitigates Fermi‐level pinning at the metal–semiconductor interface by eliminating direct metal deposition onto MoS_2_, thereby suppressing the formation of metal‐induced gap states (MIGS) and achieving cleaner electrical contacts [45]. Moreover, since the MoS_2_ layer resides atop the metal electrodes, its exposed top surface remains accessible for subsequent interface engineering, including doping and passivation treatments. Following MoS_2_ transfer, a dual‐layer gate dielectric stack (2 nm pV3D3/15 nm Al_2_O_3_) was conformally deposited. Previous reports have shown that pV3D3 effectively minimizes interfacial defect states and charge scattering, making it an ideal seed layer for top‐gated MoS_2_ FETs [27].

**FIGURE 4:**
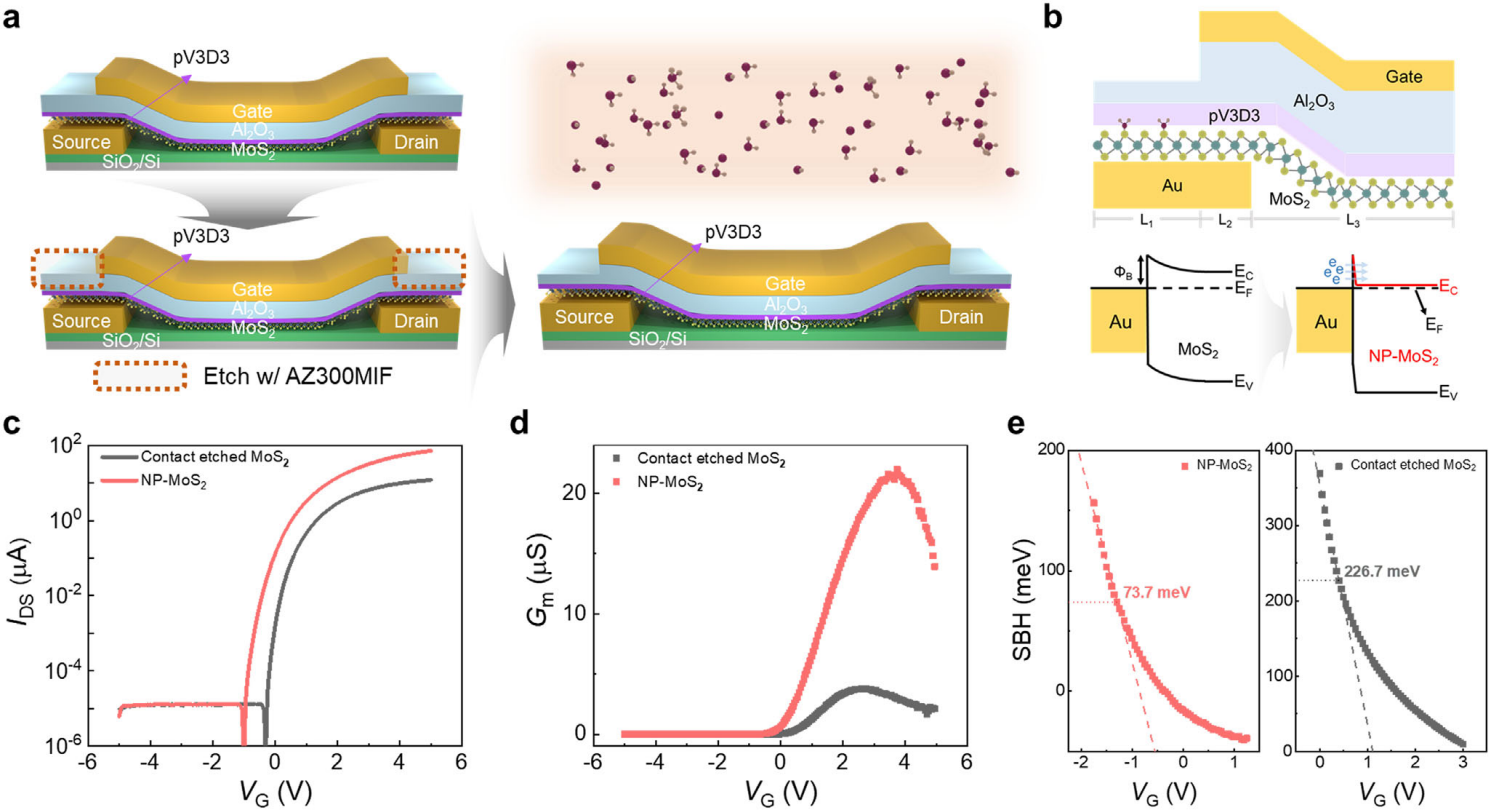
Selective contact‐region NH_3_ plasma doping in top‐gated MoS_2_ FETs. (a) Schematic of a source/drain‐first top‐gated device in which monolayer MoS_2_ is transferred onto pre‐patterned Au electrodes. (b) Cross‐sectional illustration of the NH_3_ plasma treated device structure, delineating L_1_ (plasma‐accessible contact region), L_2_ (source/drain–gate overlap region), and L_3_ (gated channel region). (c–d) Transfer and transconductance characteristics of the MoS_2_ FETs before and after NH_3_ plasma treatment. e Schottky barrier height (SBH) as a function of V_TG_ of MoS_2_ FETs before and after NH_3_ plasma treatment.

To achieve contact‐selective doping, it was essential to expose the source/drain MoS_2_ regions to the NH_3_ plasma environment. In the completed top‐gate structure, however, the dielectric layer above the contact regions was significantly thicker than our designed PD stack, which could hinder NH_2_ radicals from reaching the underlying MoS_2_ surface. Therefore, an additional thinning step of the Al_2_O_3_ layer was required to enable a controlled doping process to the contact areas. However, plasma‐based dry etching for dielectric removal risks inducing lattice damage and unintentional doping in MoS_2_ [8]. Therefore, we adopted a wet‐etching approach using AZ300 MIF, where the gate metal served as a self‐aligned mask. This method selectively removed only the excess Al_2_O_3_ above the contact regions, leaving only the 2 nm pV3D3/2 nm Al_2_O_3_ bilayer on top of the MoS_2_, which functioned as an effective PD stack during the NH_3_ plasma treatment. The AZ300MIF‐based Al_2_O_3_ etch‐rate characterization and cross‐sectional EDS verification of the etched top‐gated structure are provided in Figure S14. Interestingly, even prior to plasma exposure, a slight positive V_TH_ shift and moderate mobility enhancement were observed after Al_2_O_3_ thinning (Figure S15), which can be attributed to the removal of positively charged defect states in the etched Al_2_O_3_ layer [46, 47]

As with the preceding etching process, subsequent NH_3_ plasma treatment was performed using the gate metal as a self‐aligned mask, thereby confining doping to the MoS_2_ regions covered by the PD stack above the source and drain contacts. Our goal was to suppress unnecessary diffusion into the channel region while selectively enhancing electron injection at the contact areas. This approach aimed to improve carrier mobility without compromising the enhancement‐mode operation. Figure 4b presents a cross‐sectional schematic of the device together with the corresponding energy band diagrams before and after doping. The contact area can be conceptually divided into three regions: (1) Region L_1_, which is protected by the PD stack and is the area where the NH_3_ plasma can directly interact with the MoS_2_ surface. (2) Region L_2_, which overlaps the gate metal and is therefore not directly exposed to the plasma. Although this region does not undergo intentional plasma doping, partial carrier diffusion from L_1_ may still occur, which can contribute to reducing the contact resistance. (3) Region L_3_, the channel region located beneath the gate metal. While doping in this region can effectively reduce channel resistance, it also leads to degraded switching behavior, including a reduced on/off ratio and a pronounced deterioration in subthreshold swing (SS). In contrast, selective doping of the L_1_ region significantly lowers the Schottky barrier, thereby enhancing carrier injection while maintaining the electrostatic control of the channel [48] Therefore, our approach focuses on selective L_1_ doping, which enhances carrier injection while sustaining the switching characteristics and preserving enhancement‐mode operation of the device.

As shown in Figure 4c,d the transfer and transconductance characteristics of the device before and after doping. For accurate mobility extraction, the gate capacitance (C_ox_) of the pV3D3/Al_2_O_3_ stack was determined from V_TH_ shifts under back‐gate bias in a separate underlapped top‐gated MoS_2_ device (Figure S16), which eliminates overlap‐induced parasitic capacitances and ensures a reliable value. Based on this capacitance value, the two‐probe field‐effect mobility was extracted from the transconductance in the linear regime using the following Equation (2):

(2) $$\mu_{\mathrm{FE}}=\frac{L}{WC_{\mathrm{ox}}V_{\mathrm{DS}}}\frac{dI_{\mathrm{DS}}}{dV_{\mathrm{GS}}}$$

where *L* and *W* are the channel length and width, respectively, *C*_ox_ is the gate capacitance per unit area, *V*_DS_ is the drain voltage, and *dI*_DS_/ *dV*_GS_ is the transconductance extracted from the top‐gate transfer curve. The extracted field‐effect mobility increased by 5.8‐fold after plasma treatment. The on‐current also increased by 5.8‐fold. Importantly, the V_TH_ and on/off ratio remained nearly unchanged, confirming that the doping was confined to the contact (L_1,2_) regions with only minimal diffusion into the channel. To elucidate the physical origin of performance enhancement, we further conducted temperature‐dependent electrical measurements. As shown in Figure 4e, the effective Schottky barrier height was significantly reduced from 226.7  to 73.7 meV following plasma doping. This reduction originates from Schottky barrier thinning due to increased electron concentration in the contacts, explaining the enhanced mobility and on‐current after NH_3_ plasma treatment. The reproducibility of this contact‐doping effect was further verified by statistical analysis of 20 overlap top‐gated MoS_2_ FETs, where the distributions of SS, V_TH_, and field‐effect mobility confirmed improved carrier injection while preserving gate‐controlled switching characteristics (Figure S17 and Table S4). In addition, these results also support the uniformity of the AZ300MIF‐based Al_2_O_3_ thinning step prior to NH_3_ plasma treatment.

### 2.5 Extension Resistance Engineering in Underlapped MoS_2_ FETs

The ungated extension regions remain the bottleneck for carrier injection, especially under low‐power operation. To address the high series resistance inherent to ungated extension regions, we fabricated underlapped top‐gated MoS_2_ FETs and applied our selective NH_3_ plasma doping strategy to these areas. Figure 5a illustrates the architecture of our underlapped top‐gated MoS_2_ FETs. In this structure, the channel beneath the gate metal is efficiently modulated by the top‐gate voltage, whereas the exposed extension regions exhibit intrinsically high series resistance due to the low carrier density of MoS_2_ in these areas. However, the extension regions were initially covered with a thick Al_2_O_3_, necessitating a controlled wet‐etching step to enable subsequent NH_3_ plasma doping. Using the gate metal as a physical mask, we employed a wet etching process to thin down the extension regions, leaving behind a controlled barrier layer suitable for subsequent NH_3_ plasma doping. The actual dimensions of the fabricated device were confirmed through SEM analysis (Figure 5b), revealing extension region lengths of 694 nm and 655 nm on the left and right sides, respectively. Even though the extension regions are physically short relative to the channel length (*L*_ch_ = 12 µm), their high resistance is considered to severely limit the overall performance of the device. To examine the effect of series resistance, we compared the transfer characteristics before and after doping under varying back‐gate biases.

**FIGURE 5:**
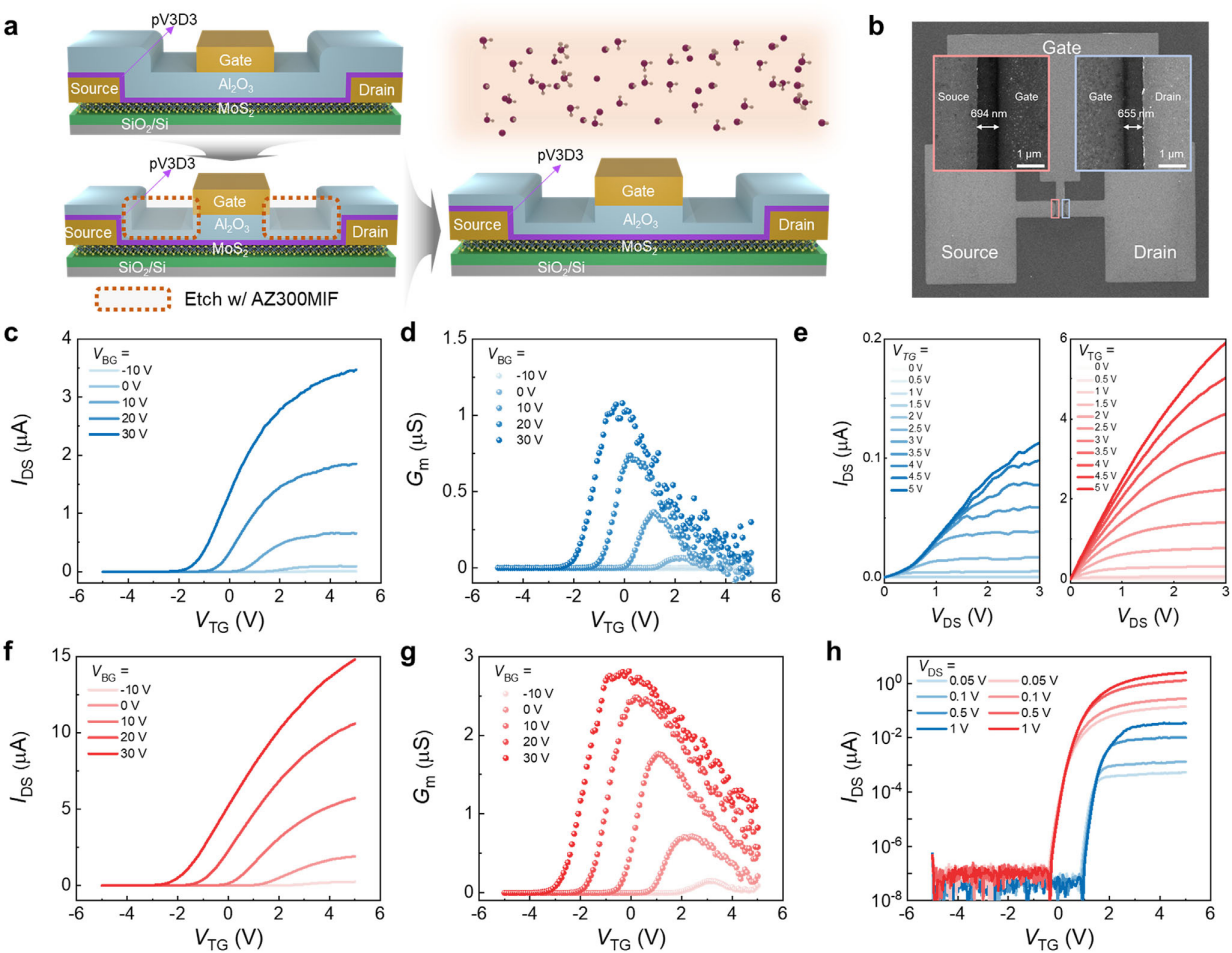
Extension‐region activation with reduction of series resistance in underlapped top‐gated MoS_2_ transistors. (a) Process flow for the fabrication of underlapped top‐gated MoS_2_ FETs. (b) SEM image of the fabricated device showing extension lengths of 694 nm and 655 nm on the left and right sides, respectively. (c–d) Transfer and transconductance characteristics of the undoped device measured under varying back‐gate biases. e Output characteristics before (blue) and after (red) extension doping. (f–g) Transfer and transconductance characteristics of the doped device measured under varying back‐gate biases. h Transfer characteristics measured at different drain biases for undoped (blue) and doped (red) devices.

In the undoped device, the high resistance of the extension regions dominates the channel at zero or negative back‐gate bias, resulting in early current saturation due to current limitation through the resistive extensions. Furthermore, when a small positive back‐gate bias (V_BG_ = 10 V) was applied, the extension resistance no longer dominated the electron transport, leading to a sharp increase in the on‐current. (Figure 5c) This result underscores the often‐overlooked impact of extension region resistance in limiting the performance of 2D FETs, particularly where current injection is most sensitive to series resistance. In contrast, the doped device maintains linear current increase over a broader gate voltage range, indicative of improved carrier injection and reduced series resistance. (Figure 5f) These trends are further confirmed by the transconductance characteristics (Figure 5d–g). While the undoped device exhibits an abrupt rise in transconductance at V_BG_ = 10 V, the doped device shows a gradual and steady increase without such a dramatic transition. This contrast confirms that selective NH_3_ plasma doping effectively activates the ungated extensions, ensuring carrier injection. Importantly, even after doping, the device retains enhancement‐mode operation without a significant shift in V_TH_. This suggests that the applied NH_3_ plasma treatment mainly affects the exposed extension regions, while unwanted diffusion into the gated channel appears to be largely suppressed. The output characteristics (Figure 5e) further highlight the distinct difference between the undoped and doped devices. The undoped device displays unstable conduction and a low on‐current resulting from limited carrier injection, while the doped device exhibits stable, linear output behavior consistent with ohmic‐like contact formation. Figure 5h further highlights the importance of extension resistance in low‐power operation by comparing the transfer curves at various drain voltages. At low V_DS_ (0.05 V), the undoped device shows rapid current saturation at very low current levels, while the doped device achieves an on‐current that is approximately 260 times higher. This confirms that sufficient doping in the extension region is essential for maintaining normal device operation under low‐power bias conditions, where series resistance becomes increasingly dominant. The fact that enhancement‐mode operation is preserved even after doping emphasizes the advantage of our method in reducing localized resistance without disrupting gate controllability. Selective extension doping effectively reduces injection‐limiting resistance while preserving strong gate control. This capability is crucial for stable top‐gated MoS_2_ FET operation without relying on global electrostatic gating. The reproducibility of this extension‐region doping effect was further confirmed by statistical analysis of 20 underlap top‐gated MoS_2_ FETs, where the distributions of SS, V_TH_, and on‐current showed consistent enhancement in current drivability and improved effective gate‐controlled switching behavior (Figure S18 and Table S5). Compared with previously reported spacer‐doping approaches, which frequently induce excessive carrier injection into the channel and lead to large V_TH_ shifts that hinder low‐power operation, our plasma‐based method enables spatially confined conductivity enhancement while preserving enhancement‐mode switching [8, 15, 49, 50]. This controllable doping profile allows the device to maintain a low‐power operating regime without relying on back‐gate electrostatic compensation. To quantitatively compare our barrier‐assisted NH_3_ plasma doping approach with previously reported doping strategies for 2D TMD transistors, key parameters including doping concentration, contact resistance, and field‐effect mobility are summarized in Table S6. Collectively, the proposed barrier‐assisted plasma doping achieves spatially confined conductivity enhancement in the underlapped access regions, mitigating the trade‐off between series resistance reduction and V_TH_ stability. By suppressing parasitic resistance without compromising gate electrostatics, the approach enables high‐performance, low‐power 2D logic and is directly compatible with scalable 2D CMOS integration.

## 3 Discussion

We have developed a barrier‐assisted NH_3_ plasma doping strategy that achieves high‐density, spatially confined n‐doping of monolayer MoS_2_ without structural degradation. A bilayer stack consisting of 2 nm pV3D3 and 2 nm Al_2_O_3_ acts as a “chemical filter”, enabling the controlled diffusion of reactive –NH_x_ radicals while blocking plasma‐induced kinetic damage. This approach yields degenerate carrier densities ($∼\;4\times 10^{13}cm^{-2}$). DFT calculations revealed a dual‐functional mechanism: while NH_2_ adsorption provides the primary donor states near the CBM, NH radicals effectively passivate sulfur vacancy defects by occupying the vacancy sites. This passivation is critical as it eliminates mid‐gap trap states and restores the pristine electronic environment of the MoS_2_ lattice. Consequently, the doped devices exhibit a transition from the defect‐dominated “hopping transport” characteristic of untreated MoS_2_ to “band‐like transport,” confirming the high electronic quality of the doped regions.

The spatial selectivity of the process allows for precise, region‐specific resistance engineering: Contact doping successfully thins the Schottky barrier (from 226.7 to 73.7 meV), enhancing field‐effect mobility and on‐current by 5.8‐fold with only negligible V_TH_ shift. Furthermore, in underlapped top‐gated devices, extension‐region doping substantially mitigates the dominant parasitic series resistance, improving low‐bias on‐current by 260‐fold (V_DS_ = 0.05 V). Notably, this performance boost was achieved while maintaining enhancement‐mode operation, which is critical for minimizing static power consumption in logic circuits. Because this method is selective, scalable, and avoids irreversible lattice damage inherent to direct plasma exposure, it provides a practical and integrable route toward high‐performance, energy‐efficient, and 3D‐compatible 2D CMOS platforms.

## 4 Experimental Method

### 4.1 MoS_2_ Synthesis and Transfer

The MoS_2_ film was synthesized within the inner quartz tube (2 inches) situated in the outer quartz tube (4 inches) furnace. High‐purity argon (Ar, 120 sccm) was used as the carrier gas under atmospheric pressure (≈750 Torr). Sulfur powder (120–180 mg, Sigma–Aldrich 99.98%) was placed in the upstream region of the furnace, while molybdenum trioxide (MoO_3_) powder (3.0–3.5 mg, Sigma–Aldrich ≥99.5%) and the Si/SiO_2_ growth substrates were positioned downstream. Note that perylene 3,4,9,10‐tetracarboxylic acid tetrapotassium salt (PTAS) was applied as a seeding promoter on the growth substrate. During heating, the upstream and downstream temperatures were raised to 830°C and 320°C, respectively, enabling sulfur vaporization, MoO_3_ reduction, and subsequent formation of monolayer MoS_2_ on the downward‐facing substrates. For transfer, polystyrene (PS) was spin‐coated onto the as‐grown MoS_2_ and soft‐baked at 85°C for 15 min to provide mechanical support. The PS/MoS_2_ stack was then delaminated from the growth substrate in deionized water and transferred onto the target substrate. After transfer, the PS layer was removed with toluene, yielding a MoS_2_ film suitable for device fabrication.

### 4.2 Device Fabrication

Si/SiO_2_ substrates were cleaned in a piranha solution (H_2_SO_4_:H_2_O_2_ = 1:1) for 20 min and rinsed thoroughly with deionized water. For the back‐gated devices, Cr (5 nm)/Au (35 nm) gate electrodes were first deposited via thermal evaporation, followed by the formation of a 15 nm thick Al_2_O_3_ bottom‐gate dielectric using ALD at 150°C. Monolayer MoS_2_ was then transferred onto the dielectric surface using a PS‐assisted wet‐transfer method. The active channel region was defined using GXR601 photoresist, and unprotected MoS_2_ was removed by O_2_ plasma etching using an ICP‐RIE system. After channel patterning, Cr (5 nm)/Au (35 nm) source and drain electrodes were deposited by thermal evaporation. The sequence between MoS_2_ transfer and S/D formation was adjusted depending on the intended doping region: MoS_2_ was transferred before S/D deposition when only the channel required doping, whereas S/D electrodes were formed first when the doping needed to extend into the contact regions. Following electrode formation, a PD stack was deposited on the device. First, a 2 nm pV3D3 layer was grown by iCVD using V3D3 (300 sccm) and TBPO (100 sccm) at a substrate temperature of 40°C and a filament temperature of 250°C. After this step, an additional 2 nm Al_2_O_3_ capping layer was deposited by ALD at 150°C. Top‐gate devices were fabricated using a similar process flow, with the primary distinction being the relative order of the MoS_2_ channel and the S/D electrodes. In overlapped top‐gate structures, Cr (5 nm)/Au (35 nm) S/D electrodes were patterned on the Si/SiO_2_ substrates prior to MoS_2_ transfer. In underlapped top‐gate structures, MoS_2_ was first transferred onto the substrate, and the S/D electrodes were deposited afterward. After defining the MoS_2_ channel, the top‐gate dielectric was formed by sequential deposition of 2 nm pV3D3 (iCVD) and 15 nm Al_2_O_3_ (ALD, 150°C). Finally, Cr (5 nm)/Au (35 nm) gate electrodes were patterned using NR9 photoresist and deposited by thermal evaporation.

### 4.3 Electrical and Optical Properties Characterization

A parameter analyzer (Keithley 4200 SCS) integrated with a probe station (MS‐TECH, MST‐1000B) was used for standard electrical characterization, whereas a high‐resolution source measurement unit (Keysight B1500A) enabled precision measurements. All electrical tests were performed under dark conditions and in vacuum (≈5 × 10^−2^ Torr) to suppress environmental effects. For in‐house micro‐photoluminescence (PL) and Raman spectroscopy, scattered light was collected using a spectrometer (Andor Kymera 328i) equipped with a charge‐coupled device detector (Andor iDus DU401ABVF). A 532 nm laser was used as the excitation source for both PL and Raman measurements, and identical exposure times were employed across samples to ensure consistent intensity comparisons.

### 4.4 Computational Method

We performed DFT calculations using the Vienna Ab initio Simulation Package (VASP) based on the projected augmented wave method [51, 52]. Plane waves were expanded with a kinetic energy cutoff of 400 eV with the self‐consistency energy criterion of 10^−4^ eV. Atomic structures were optimized using conjugate‐gradient approach until the Hellmann–Feynman forces were less than 0.01 eV/Å. The simulations were performed within the Perdew‐Burke‐Ernzerhof form of generalized gradient approximation [53] with weak long‐range dispersion interactions corrected within the DFT‐D3 scheme [54]. To model 2H MoS_2_ monolayer, we adopted a 5 × 5 supercell with 30 Å vacuum spacing Monkhorst‐pack k‐point grid of 4 × 4 × 1 was employed for structural relaxation and 12 × 12 × 1 for density of states calculation. Further computational details can be found in Refs [55, 56].

## Author Contributions

I. L. conceived the research, designed the experiments, carried out device fabrication and processing, and performed the electrical characterization with assistance from S. P., M. L., and H. L. J.P. and S.Y. performed the density functional theory calculations and contributed to the theoretical analysis. S.Yo. assisted with figure preparation. D. K., K. R., S. K., K. K., and W. A. contributed to device fabrication and participated in data discussion and analysis. K. Ka. provided guidance on manuscript preparation and contributed to the scientific interpretation. I.L. and J.P. wrote the manuscript with input and discussion from all authors. S.‐Y. C. supervised the overall project and Y.‐H. K. oversaw the computational part and they provided critical feedback and revisions to the manuscript. All authors discussed the results and reviewed the manuscript.

## Conflicts of Interest

The authors declare no conflicts of interests.

## Supporting information




**Supporting File**: adma74389‐sup‐0001‐SuppMat.docx.

## Data Availability

Relevant data supporting the key findings of this study are available within the article and the Supplementary Information file. All raw data generated during the current study are available from the corresponding authors upon request.
