# Supplementary material for: Barrier‐Assisted Plasma Doping for Spatially Selective Resistance Engineering in MoS2 Transistors
Source: Adv Mater. 2026 Aug 1;38(55):e74389. doi: 10.1002/adma.74389 (PMC13629280; doi:10.1002/adma.74389)
Supplement: Supplementary file 1 [file ADMA-38-e74389-s001.docx]

Barrier-Assisted Plasma Doping for Spatially Selective Resistance Engineering in MoS_2_ Transistors

Inseong Lee^1^, Joonho Park^1^, Seungsun Yoo^2^, Seunghyun Yu^1^, Mingu Kang^1^, Seohak Park^1^, Min Kyu Lee^1^, Hyeongjin Lim^1^, Dongyoung Kim^3^, Kun Woo Roh^2^, Sejin Kim^1^, Kihyuk Kim^1^, Wonbae Ahn^1^, Kibum Kang^2,3*^, Yong-Hoon Kim^1,2*^, and Sung-Yool Choi^1,2*^

^1^School of Electrical Engineering, Korea Advanced Institute of Science and Technology (KAIST), 291 Daehak-ro, Yuseong-gu, Daejeon 34141, Republic of Korea

^2^Graduate School of Semiconductor Technology, Korea Advanced Institute of Science and Technology (KAIST), 291 Daehak-ro, Yuseong-gu, Daejeon 34141, Republic of Korea

^3^Department of Materials Science and Engineering, Korea Advanced Institute of Science and Technology (KAIST), 291 Daehak-ro, Yuseong-gu, Daejeon 34141, Republic of Korea

^*^Corresponding author

Correspondence to

*Sung-Yool Choi* ([sungyool.choi@kaist.ac.kr](mailto:sungyool.choi@kaist.ac.kr))

*Yong-Hoon Kim* ([y.h.kim@kaist.ac.kr](mailto:y.h.kim@kaist.ac.kr))

*Kibum Kang* ([kibumkang@kaist.ac.kr](mailto:kibumkang@kaist.ac.kr))

**KEYWORDS:** Molybdenum disulfide, 2D transistor, NH_3_ plasma, Protective dielectric stack, Degenerate doping, Enhancement-mode

Figure S1. Optical microscopy and Raman characterization of pV3D3-deposited MoS_2_ before and after NH_3_ plasma treatment.
a–b Optical microscopy images of the pV3D3-deposited MoS_2_ surface taken before and after NH_3_ plasma exposure. After plasma treatment, the MoS_2_ grains exhibit a noticeably lighter contrast, indicating that the pV3D3 layer did not fully protect the underlying MoS_2_ and that partial etching occurred. c Raman spectra of the same regions, showing severe degradation of crystallinity after plasma treatment. Both the E_2_g and A_1_g modes display significantly reduced intensities and pronounced peak broadening, confirming substantial structural damage to the MoS_2_ lattice.

Figure S2. AFM analysis of dielectric deposition on MoS_2_.
a 3D AFM topography of MoS_2_ after direct deposition of a 2 nm Al_2_O_3_ layer, showing non-uniform surface morphology and increased roughness (R_q_ = 0.39 nm). b 3D AFM topography of MoS_2_ with a 2 nm pV3D3 interfacial layer followed by 2 nm Al_2_O_3_. The introduction of pV3D3 yields a much more uniform morphology and significantly reduced roughness (R_q_ = 0.13 nm), confirming its role in improving dielectric nucleation and film conformality.

Figure S3. Raman spectroscopy characterization of MoS_2_ after NH_3_ plasma treatment. MoS_2_ with the protective-dielectric (PD) deposited retains distinct E_2g_ and A_1g_ vibrational modes, indicating preserved crystallinity (red). In contrast, MoS_2_ without the PD exhibits severe structural degradation, resulting in the complete absence of identifiable MoS_2_ Raman features (blue).

Figure S4. XPS characterization of before and after NH_3_ plasma treatment through the PD. NH_3_ plasma-treated MoS_2_ (NP-MoS_2_) exhibits a 0.3 eV blue shift in the Mo 3d peaks, indicating an increase in electron density. This shift provides clear evidence of n-type doping induced by the NH_3_ plasma treatment.

Figure S5. HAADF image and EDS elemental mapping of NP-MoS_2_. The N signal is clearly observed overlapping the S and Mo regions, confirming the presence of nitrogen adsorbed at the MoS_2_ surface.

Figure S6. Preliminary verification of NH_3_ plasma doping in monolayer WSe_2_ FETs.
a Transfer characteristics of a pristine monolayer WSe_2_ FET, showing typical p-type transport behavior with dominant hole conduction under negative gate bias. b Transfer characteristics of the NH_3_-plasma-treated WSe_2_ FET, showing dominant electron conduction under positive gate bias. The polarity conversion from p-type to n-type behavior suggests that the NH_3_ plasma doping effect may be extendable beyond MoS_2_ to other 2D TMD semiconductors.

**Figure S7. The model for MoS_2_ PDOS calculation.
a** $5\times5$ unit cell model of 2H MoS_2_ was employed for NH_x_ surface adsorption and vacancy model. When one NH_2_ radical is adsorbed on S atom, its surface density becomes $4.7\times{10}^{13}cm^{-2}$. **b** One S atom was eliminated for defect calculation. The circled yellow region denotes the S atom vacancy site (V_S_).

**Figure S8. Spin-polarized projected density of states (PDOS) of NH_2_/MoS_2_ and NH_2_/MoS_2_-V_S_ and their partial charge densities.
a** In PDOS, the spin-up and spin-down eigenvalues are nearly degenerate for MoS_2_-derived bands, with an energy difference of approximately 0.02 eV, indicating negligible spin polarization of the substrate. In contrast, the defect state near the Fermi level exhibits pronounced exchange splitting of ~0.16 eV. This demonstrates that the spin polarization originates from the NH_2_ moiety with an odd number of electrons, while the MoS_2_ substrate remains essentially non-magnetic. **b** The partial charge density is calculated for the occupied defect state just below Fermi level (denoted as arrow in **a**), which shows that charges are more distributed on MoS_2_ substrate than NH_2_ moiety. **c** In PDOS of NH_2_/MoS_2_-V_S_, the Fermi level remains shifted toward the conduction-band region, indicating that NH_2_ trapped at the sulfur vacancy still induces n-type characteristics. The spin polarization calculation is stabilized by 0.14 eV compared with the nonmagnetic calculation where the defect states are split by ~ 0.56 eV. **d** The partial charge density of the occupied defect states is distributed more strongly in MoS_2_ substrate, even though the state originates from the NH_2_ moiety.

**Figure S9. Projected density of states (PDOS) for various MoS_2_ derivatives.
a** Density of states **(**DOS) of a pristine MoS_2_ monolayer. Energies are referenced to the valence band maximum (VBM) denoted as solid vertical line. **b** PDOS of MoS_2_-NH where the NH group is adsorbed on a sulfur site. An N-induced state emerges near VBM. **c** A sulfur vacancy (V_S_) introduces defect states within the bandgap. Yellow circle in the inset shows the S-vacancy site. **d** Upon passivation of the V_S_ site by N substitution (N_S_), the defect state shifts toward the VBM, indicating acceptor-like defect behavior. The dotted vertical line denotes Fermi level and the yellow circle in the inset shows N_S_ site.

**Figure S10. Temperature-dependent PL analysis of defect-related localized emission in MoS_2_ before and after NH_3_ plasma treatment.
a–b** Temperature-dependent PL spectra of pristine MoS_2_ and NP-MoS_2_ measured from 77 to 277 K. The spectra are vertically offset for clarity. **c** Extracted intensity ratio of the localized emission peak to the A peak, *I*_L_/*I*_A_, as a function of temperature. The reduced *I*_L_/*I*_A_ ratio after NH_3_ plasma treatment indicates suppression of defect-related localized emission. **d** Extracted A peak positions as a function of temperature. The red-shifted A peak after NH_3_ plasma treatment is consistent with increased electron density and n-type doping in MoS_2_.

Figure S11. Statistical analysis of BG MoS_2_ FETs after NH_3_ plasma doping.
a Transfer characteristics of BG MoS_2_ FETs in the pristine state, after channel doping, and after channel and contact doping (Dual doping). b-c Box plots of the extracted threshold voltage and field-effect mobility from 20 BG devices for each condition. The statistical distributions confirm the reproducible threshold-voltage modulation and field-effect mobility enhancement induced by NH_3_ plasma doping through the PD stack. The corresponding quantitative values are summarized in Table S3.

**Figure S12. Stability evaluation of NH_3_ plasma doped BG MoS_2_ FETs.
a** Transfer characteristics of the NH_3_ plasma doped BG MoS_2_ FET measured over 4 weeks under ambient conditions and after annealing at 250 °C. **b–c** Transfer characteristics measured under positive-bias stress (PBS) and negative-bias stress (NBS), respectively. The nearly unchanged transfer characteristics in all cases indicate negligible variation in the doping level and on-current, confirming the environmental, thermal, and electrical stability of the NH_3_ plasma induced doping effect.

Figure S13. Temperature-dependent transfer curves of undoped devices. The device exhibits insulating behavior, characterized by an increase in conductivity with increasing temperature. This trend is attributed to hopping transport mediated by intrinsic defect states such as sulfur vacancies and interfacial traps.

**Figure S14. Etch-rate characterization and structural verification of the AZ300MIF-based Al_2_O_3_ wet-etching process for top-gated MoS_2_ FETs.**
**a** Removed Al_2_O_3_ thickness as a function of AZ300MIF etching time. **b** Linear fitting of the average removed Al_2_O_3_ thickness values. This extracted etch rate was used to determine the Al_2_O_3_ thinning condition for the top-gated devices. **c** Cross-sectional EDS mapping of the top-gated MoS_2_ FET after AZ300MIF etching. The reduced Al signal in the exposed region compared with the unetched gate-covered region confirms selective thinning of Al_2_O_3_. The remaining Al signal in the etched region indicates that the residual Al_2_O_3_ barrier is preserved, thereby maintaining the protective pV3D3/Al_2_O_3_ stack for subsequent NH_3_ plasma doping.

Figure S15. Transfer characteristics of the MoS_2_ FETs before (blue) and after (black) Al_2_O_3_ etching at the contact region. A slight positive shift in the threshold voltage and an improvement in mobility are observed after etching, which can be attributed to the reduction of defect states, such as fixed oxide charges, originally present in the Al_2_O_3_ layer.

Figure S16. Threshold voltage (V_TH_) from the top gate sweep as a function of the applied back-gate voltage (V_BG_) from NH_3_ plasma treated underlap top-gated MoS_2_ FET. The top-gate dielectric consists of 2 nm pV3D3 and 15 nm Al_2_O_3_. The slope in this figure corresponds to the ratio between the top-gate EOT and the back-gate EOT. Using the 90 nm SiO_2_ back-gate dielectric as a reference, the extracted top-gate EOT is estimated to be approximately 12.8 nm.

**Figure S17. Statistical analysis of overlap top-gated MoS_2_ FETs after Al_2_O_3_ etching and NH_3_ plasma doping.** **a** Transfer characteristics of overlap top-gated MoS_2_ FETs in the pristine state, after Al_2_O_3_ etching, and after NH_3_ plasma doping. **b–d** Box plots of the extracted SS, V_TH_, and field-effect mobility from 20 overlap TG devices for each condition. The statistical distributions confirm that the AZ300MIF-based Al_2_O_3_ etching and NH_3_ plasma doping processes reproducibly enhance carrier injection while preserving gate-controlled switching characteristics. The corresponding quantitative values are summarized in Table S4.

**Figure S18. Statistical analysis of underlap top-gated MoS_2_ FETs after Al_2_O_3_ etching and NH_3_ plasma doping.
a** Transfer characteristics of underlap top-gated MoS_2_ FETs in the pristine state, after Al_2_O_3_ etching, and after NH_3_ plasma doping. **b–d** Box plots of the extracted SS, threshold voltage and on-current from 20 underlap TG devices for each condition. The statistical distributions confirm that selective extension-region doping reproducibly enhances current drivability while maintaining gate-controlled switching characteristics by reducing the parasitic series resistance of the ungated extension regions. The corresponding quantitative values are summarized in Table S5.

**Table S1.** Stepwise radical reaction energies on top of S and V_S_ sites plotted on Figure 2. Formation energy (E_form_) of the system can be obtained from summation of the stepwise radical reaction energies. All the systems with odd number of electrons are calculated with spin polarization.

| E_react, radical_ [eV]/  E_form_ [eV] | N | + H (NH) | + H (NH_2_) |
| --- | --- | --- | --- |
| On top of S | -1.70  -1.70 | -4.28  -5.98 | -2.53  -8.50 |
| V_S_ | -6.19  -6.19 | -4.07  -10.27 | -1.02  -11.29 |

**Table S2.** Binding energy of NH_x_ radicals on top of S of a pristine MoS_2_ and V_S_ sites.

| Binding Energy [eV] | N | NH | NH_2_ |
| --- | --- | --- | --- |
| On top of S | -1.70 | -2.14 | -0.33 |
| V_S_ | -6.19 | -6.43 | -3.11 |

**Table S3. Statistical summary of BG MoS_2_ FET characteristics.** Quantitative statistical values of V_TH_ and field-effect mobility extracted from 20 BG MoS_2_ FETs for each condition: pristine, channel doping, and dual doping.

| Condition | Pristine | Channel doping | Dual doping |
| --- | --- | --- | --- |
| V_TH_ (V) | 0.39 ± 0.28 | -13.79 ± 3.51 | -14.24 ± 1.22 |
| Mobility (cm^2^/Vs) | 8.09 ± 2.96 | 10.91 ± 2.82 | 43.19 ± 3.90 |

**Table S4. Statistical summary of overlap top-gated MoS_2_ FET characteristics.** Quantitative statistical values of SS, V_TH_, and field-effect mobility extracted from 20 overlap top-gated MoS_2_ FETs for each condition: pristine, after Al_2_O_3_ etching, and after NH_3_ plasma doping.

| Condition | Pristine | Al_2_O_3_ Etch | NH_3_ plasma |
| --- | --- | --- | --- |
| SS_avg_ (mV/dec) | 122.98 ± 18.72 | 114.66 ± 14.93 | 116.28 ± 13.54 |
| V_TH_ (V) | -0.06 ± 0.18 | 0.07 ± 0.18 | -0.11 ± 0.21 |
| Mobility (cm^2^/Vs) | 3.15 ± 2.08 | 3.59 ± 2.25 | 20.71 ± 7.72 |

**Table S5. Statistical summary of underlap top-gated MoS_2_ FET characteristics**. Quantitative statistical values of SS, V_TH_, and on-current extracted from 20 underlap top-gated MoS_2_ FETs for each condition: pristine, after Al_2_O_3_ etching, and after NH_3_ plasma doping.

| Condition | Pristine | Al_2_O_3_ Etch | NH_3_ plasma |
| --- | --- | --- | --- |
| SS_avg_ (mV/dec) | 136.06 ± 24.49 | 135.35 ± 13.53 | 114.77 ± 19.10 |
| V_TH_ (V) | 1.14 ± 0.47 | 1.94 ± 0.38 | 0.29 ± 0.30 |
| *I*_on_ (A) | (2.87 ± 1.80)$\times$10^-7^ | (1.44 ± 0.70)$\times$10^-7^ | (3.25 ± 2.21)$\times$10^-6^ |

**Table S6. Benchmarking comparison of doping strategies for 2D TMD transistors.** Comparison of representative doping strategies for 2D TMD transistors, including the doping method, channel material, doping carrier concentration, contact resistance, and field-effect mobility.

| TMDC | Doping strategy | Doping n_2D_ (cm^-2^) | Undoped R_C_ (kΩ·μm) | Doped R_C_ (kΩ·μm) | Mobility (cm^2^/Vs) | Reference |
| --- | --- | --- | --- | --- | --- | --- |
| MoS_2_ (CVD) | Barrier-assisted  NH_3_ plasma | 4.3$\times$10^13^ | 47 | 1.45 | 52.83 | This work |
| MoS_2_ (CVD) | SiO_x_ | 1.4$\times$10^13^ | 49 | 4.5 | 44 | [1] |
| MoS_2_ (CVD) | Tetramethylammonium hydroxide (TMAH) / Acetone | 4.1$\times$10^13^ | 6.9 | 1.2 | 12 | [2] |
| MoS_2_ (CVD) | AlO_x_ with  N_2_ annealing | 2.2$\times$10^13^ | 0.74 | 0.48 | 33.5 | [3] |
| MoS_2_ (Flake) | *N*-methyl-pyrrolidone (NMP) | 1.6$\times$10^13^ | 87 | 21.2 | 22.2 | [4] |
| MoS_2_ (CVD) | Benzyl Viologen (BV) | 1.2$\times$10^13^ | 3.3 | 1.1 | 24.7 | [5] |
| MoS_2_ (MOCVD) | Reverse sputtering | 4.3$\times$10^12^ | 1126 | 432 | 1.2 | [6] |
| MoS_2_ (CVD) | HfO_x_ | 1.2$\times$10^13^ | - | ~5 | 50 | [7] |
| WSe_2_ (CVD) | Chloroform (CHCl_3_) | 1.8$\times$10^13^ | 168 | 2.5 | 36.2 | [8] |
| WSe_2_ (Flake) | α-RuCl_3_ | 3.1$\times$10^13^ | - | 4 | 21 | [9] |
| WSe_2_ (CVD) | HAuCl_4_ | 1.81$\times$10^13^ | - | 0.81 | 95.03 | [10] |
| WSe_2_ (CVD) | NO annealing | 1.77$\times$10^13^ | - | 1.3 | 16.1 | [11] |

**Reference**

1. P.-H. Ho, et al., “High-Performance Two-Dimensional Electronics with a Noncontact Remote Doping Method,” *ACS Nano* **17** (2023): 12208–12215, doi: 10.1021/acsnano.3c00522.
2. P.-H. Ho, et al., “Hysteresis-Free Contact Doping for High-Performance Two-Dimensional Electronics,” *ACS Nano* **17** (2023): 2653–2660, doi: 10.1021/acsnano.2c10631.
3. C. J. McClellan, et al., “High Current Density in Monolayer MoS_2_ Doped by AlO_x_,” *ACS Nano* **15** (2021): 1587–1596, doi: 10.1021/acsnano.0c09078.
4. X.-K. Li, et al., “Controllable Doping of Transition-Metal Dichalcogenides by Organic Solvents,” *Advanced Electronic Materials* **6** (2020): 1901230, doi: 10.1002/aelm.201901230.
5. D. Kiriya, et al., “Air-Stable Surface Charge Transfer Doping of MoS_2_ by Benzyl Viologen,” *Journal of the American Chemical Society* **136** (2014): 7853–7856, doi: 10.1021/ja5033327.
6. Y. Fa, et al., “Contact Resistance Optimization in MoS_2_ Field-Effect Transistors through Reverse Sputtering-Induced Structural Modifications,” *ACS Applied Materials & Interfaces* **17** (2025): 24526–24534, doi: 10.1021/acsami.4c21596.
7. A. Alharbi and D. Shahrjerdi, “Analyzing the Effect of High-k Dielectric-Mediated Doping on Contact Resistance in Top-Gated Monolayer MoS_2_ Transistors,*” IEEE Transactions on Electron Devices* **65** (2018): 4084–4092, doi: 10.1109/TED.2018.2866772.
8. L. Hoang, et al., “Low Resistance p-Type Contacts to Monolayer WSe_2_ through Chlorinated Solvent Doping,” *Nature Communications* **17** (2026): 718, doi: 10.1038/s41467-025-65604-3.
9. J. Xie, et al., “Low Resistance Contact to P-Type Monolayer WSe_2_,” *Nano Letters* **24** (2024): 5937–5943, doi: 10.1021/acs.nanolett.3c04195.
10. P.-H. Ho, et al., “High-Performance WSe_2_ Top-Gate Devices with Strong Spacer Doping,” *Nano Letters* **23** (2023): 10236–10242, doi: 10.1021/acs.nanolett.3c02757.
11. S. Ghosh, et al., “High-Performance p-Type Bilayer WSe_2_ Field Effect Transistors by Nitric Oxide Doping,” *Nature Communications* **16** (2025): 5649, doi: 10.1038/s41467-025-59684-4.
